# Roles of Tight Junction Proteins in Intestinal Barrier Function and Health of Weaned Piglets: A Review

**DOI:** 10.3390/vetsci13020131

**Published:** 2026-01-29

**Authors:** Shijia Zhang, Guosheng Zhang, Jiqiu Xu, Danni Chen, Chenggang Yin, Jing Wang, Xianren Jiang, Chengwei Wang

**Affiliations:** 1 College of Life Sciences, Jiangxi Science and Technology Normal University, Nanchang 330013, China; shijzhang@126.com (S.Z.); dannie_chen1107@163.com (D.C.); 2 Key Laboratory of Feed Biotechnology, Ministry of Agriculture and Rural Affairs, Institute of Feed Research, Chinese Academy of Agricultural Sciences, Beijing 100081, China; ycg0701@126.com; 3 Jiangxi Agricultural Technology Extension Center, Nanchang 330046, China; 122527802@163.com; 4 Animal Husbandry and Fishery Service Center of Miluo City, Miluo 414400, China; xujiqiu888@126.com; 5 Institute of Animal Husbandry and Veterinary Medicine, Beijing Academy of Agriculture and Forestry Sciences, Beijing 100097, China

**Keywords:** tight junction proteins, weaned piglets, intestinal characterization, intestinal barrier

## Abstract

Growth stagnation or weight loss is prevalent in weaned piglets, with post-weaning diarrhea severely compromising intestinal health, underscoring the criticality of intestinal structural integrity for preventing digestive disorders. Tight junction proteins (TJPs), core components of intestinal intercellular junctions, play indispensable roles in maintaining barrier function, nutrient absorption, and intestinal homeostasis by regulating paracellular permeability and mediating immune defense-related signaling pathways. Dysregulated TJP expression disrupts these processes and impairs piglet growth performance. Despite recent progress in characterizing TJP-mediated regulation of intestinal health in weaned piglets, key knowledge gaps remain regarding the specific regulatory mechanisms by which distinct TJP subtypes modulate intestinal microbiota–immune crosstalk. Future research should prioritize elucidating the effects of nutritional interventions on TJP expression and intestinal health, as well as the molecular mechanisms underlying TJP involvement in intestinal diseases. These efforts will provide theoretical support for developing novel feed additives and nutritional strategies to improve weaned piglet health.

## Simple Summary

Growth stagnation or weight loss is common in weaned piglets during their growth and development, and post-weaning diarrhea poses a major challenge to their intestinal health. This study examined the molecular structure and functional mechanisms of tight junction proteins (TJPs) and their roles in intestinal barrier function and immune regulation in weaned piglets. Altered expression of these key intercellular proteins impairs intestinal barrier integrity and nutrient absorption, further compromising piglet growth performance. This review summarizes the important effects of tight junction proteins on piglet intestinal health, emphasizing that protecting intestinal health is essential for preventing digestive diseases, enhancing animal welfare, and improving breeding economic benefits.

## 1. Introduction

In the field of pig breeding, intestinal health problems in piglets, especially intestinal diarrhea, are a key factor affecting breeding efficiency. During the weaning period, the intestinal structure and function of piglets are not yet fully developed, so they are extremely sensitive to stress factors such as external environmental stress, infections, and dietary changes, and weaning stress may trigger a variety of physiological and behavioral responses [1]. For instance, oxidative stress is highly prevalent in weaned piglets, and oxidative stress within the intestinal lumen disrupts the oxygen-sensitive ecological niche of the gut microbiota, thereby inducing ecological dysregulation of the intestinal microbiota [2]. Importantly, such dysregulation exacerbates intestinal oxidative damage and inflammatory responses, both of which directly compromise the structural and functional integrity of TJPs—the essential structural components of the intestinal epithelial barrier [3]. Additionally, early-weaned piglets commonly display inadequate digestive enzyme secretion in the gastrointestinal tract, leading to limited digestion of solid feed [4]. The accumulation of undigested nutrients within the intestinal lumen induces osmotic imbalance and local inflammation; these stress factors not only impair the intestinal physical barrier, with TJP disruption as a primary critical event, coupled with decreased mucin secretion and increased intestinal permeability, but may also further trigger an imbalance in the intestinal microbiota [5]. Given that TJP dysfunction is a converging downstream consequence of various post-weaning intestinal disturbances, and that restoring TJP integrity is a crucial strategy to alleviate intestinal barrier damage and enhance piglet growth performance, TJPs are deemed essential for sustaining the growth and health of weaned piglets. TJPs are the main form of connection between intestinal epithelial cells and play a decisive role in maintaining the mechanical barrier function and selective permeability of the intestinal mucosal epithelium [6]. Changes in the permeability of TJPs and their structural incompleteness may lead to the penetration of substances such as microorganisms and pathogens in the intestinal tract, affecting their expression and distribution. These changes not only interfere with the regulation of intercellular transepithelial resistance but may also adversely affect the absorption of nutrients, the construction of immune barriers, and the colonization and growth of intestinal flora in piglets.

This review aims to (i) systematically analyze and integrate the current research progress on the molecular structure and function of TJPs and their effects on the intestinal barrier of weaned piglets; (ii) critically assess the extent to which the health and functional integrity of TJPs in the piglet intestine are essential for the prevention and treatment of digestive diseases and contribute to the improvement of animal welfare and the production economy of animal husbandry; and (iii) to provide a basis for guiding future scientific research and to offer valuable insights that facilitate the translation of TJP research findings into practical applications within animal nutrition research and the feed additive industry.

## 2. TJPs Overview

### 2.1. Molecular Structure of TJPs

**Figure 1:**
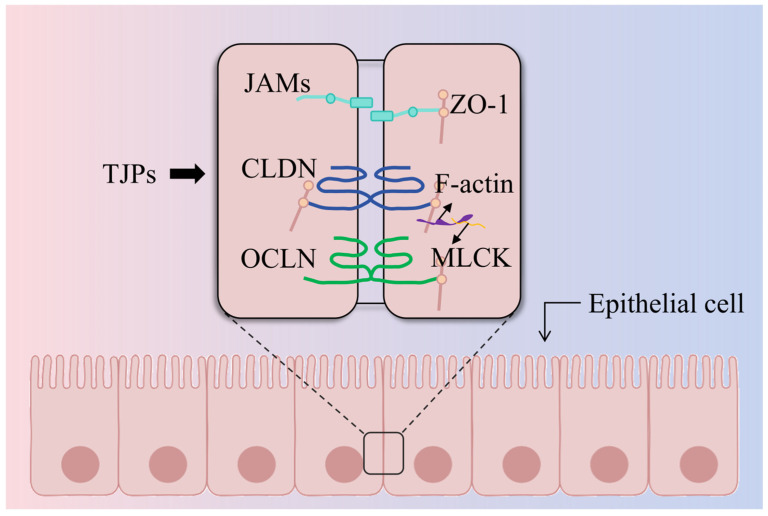
**Molecular structure of tight junction proteins (TJPs).** The TJ complex consists of transmembrane proteins, including OCLN, CLDN, and JAMs, that seal the paracellular space. These proteins are anchored to the actin cytoskeleton via scaffolding proteins such as ZO-1 to maintain structural stability. OCLN: occludin; CLDN: claudins; JAMs: junctional adhesion molecules; ZO-1: zona occludens-1; F-actin: fibrillar actin; MLCK: myosin light chain kinase.

TJPs, including occludin (OCLN), claudins (CLDN), junctional adhesion molecules (JAMs), and zona occludens (ZO) proteins, form the tight junction (TJ) complex, which is essential for the intestinal epithelial barrier [7,8]. ZO-1, ZO-2, and ZO-3 facilitate the association of transmembrane proteins, such as OCLN, CLDN, and JAMs, with the cytoskeleton. Interacting with one another on the apical surfaces of neighboring cells, they are essential in sealing the intercellular spaces (Figure 1).

OCLN is involved in intercellular adhesion and signal transduction and is one of the earliest identified TJ transmembrane proteins [7]. OCLN is a transmembrane integral membrane protein of approximately 65 kDa with four transmembrane domains, which form two extracellular loops and a long cytoplasmic tail; it is a classical TJ-associated molecule localized at the TJ of epithelial and endothelial cells [8,9]. This configuration enables its interaction with the cytoskeleton and adjacent cells, which is crucial for sealing the paracellular pathway and regulating intestinal permeability. The phosphorylation of serine, threonine, and tyrosine residues in occludin’s carboxy-terminus enhances its binding to the ZO-1’s PDZ domain. This is essential for maintaining connections between epithelial cells and participating in the transmission of cell signaling pathways. Studies have shown that the localization of OCLN within TJs is primarily determined by its level of phosphorylation [10]. The transmembrane structure of OCLN allows it to play an important role in maintaining intercellular adhesion, permeability, and regulating transepithelial electrical resistance (TEER). Under certain pathological conditions, when the structure and function of tight junctions are disrupted, and paracellular permeability is increased, OCLN can serve as an important indicator to measure changes in the structure of tight junctions [11].

CLDNs are core components of TJPs and typical cell–cell adhesion transmembrane proteins, with a critical regulatory role in TJ paracellular permeability. Structurally, all members of the CLDN family feature four transmembrane helices (four transmembrane domains), a short intracellular amino (NH_2_) terminus, an elongated intracellular carboxyl (COOH) terminus, and two extracellular segments [12,13]. The carboxy-terminal region of CLDN is instrumental in signal transduction, featuring specific binding sites that facilitate interactions with cytoplasmic tight junction proteins, such as ZO-1 and ZO-2. Studies have shown [11] that the interaction between CLDN proteins and ZO-1 can indirectly link CLDN proteins to the cytoskeletal protein actin through ZO-1. It is precisely because of the connecting role between CLDN and ZO-1, as well as actin, that the stability and selective permeability of TJs are ensured. The type and quantity of CLDN determine the permeability of the barrier, and different types of CLDN have different expression patterns and functions in different barriers. Various proteins within the CLDN family can affect paracellular permeability, thereby influencing the transport of Ca^2+^ [14,15].

JAMs belong to the immunoglobulin superfamily and are proteins located at the tight junctions of polarized epithelial cells, regulating intercellular adhesion and permeability. JAM-A, featuring a single transmembrane segment, two extracellular loops, and an immunoglobulin-like domain, which enables it to mediate cell adhesion and signaling, is crucial for tight junction assembly and regulates paracellular permeability and cell migration [16]. JAM-A often exists in homodimeric form in epithelial cells, mediating the transmission of external signals to the interior of the cell [17]. Upon homodimerization, JAM-A interacts with ZO-1, initiating a cascade that augments ZO-1 expression and contributes to the assembly of TJ complexes in conjunction with cytoskeletal proteins. This is essential for the structural integrity of various cell types, with specific expression patterns across tissues and cells [18].

ZO proteins are intracellular belt-like closure proteins that link actin filaments and transmembrane proteins and are one of the structural proteins that constitute the TJ of epithelial cells. They are widely distributed in tissues and organs such as the intestine, vascular endothelium, and renal tubules, and play an important regulatory role in processes such as transepithelial transport of substances, maintenance of polarity, cell proliferation, and differentiation, and tumor cell metastasis [19]. There are mainly three isoforms of this protein: ZO-1, ZO-2, and ZO-3, which function as heteropolymers to form a complex. In terms of cytoskeletal connections, ZO-1 binds to fibrillar actin (F-actin) to connect the surrounding actin cytoskeleton. In barrier regulation, when myosin light chain kinase (MLCK) is activated, it increases TJ permeability by contracting or relaxing the actin-myosin ring. The N-terminal region of TJPs facilitates interactions with diverse transmembrane proteins, while the C-terminal domain engages with the actin cytoskeleton and associated proteins, thereby anchoring the TJ complex to the cytoskeleton and ensuring its stability [20,21].

### 2.2. Functional Mechanisms of TJPs

TJPs are indispensable for the structural and functional integrity of intercellular tight junctions. They regulate substance permeability and modulate immune signaling, which are crucial for cellular homeostasis and immune defense.

TJPs are pivotal in upholding the cellular barrier’s integrity by establishing a physical seal at the TJ between epithelial cells [22,23]. This strategic positioning empowers TJPs to constrain the unregulated paracellular movement of macromolecules and cells, thus ensuring the selective permeability indispensable for maintaining tissue integrity and function. OCLN molecules assemble into linear configurations on the plasma membrane through their transmembrane segments, and by interacting with corresponding extracellular domains of OCLN molecules in adjacent cells, they form a closed barrier [17]. Studies have found that OCLN is a critical intestinal tight junction protein in preweaning piglets, and its elevated intestinal abundance following EA supplementation preserves intestinal barrier integrity and mitigates maternal heat stress-induced intestinal damage in piglets [24]. ZO proteins connect TJs to the internal structure of cells by linking to the actin cytoskeleton [25]. In studies of embryoid bodies rendered ineffective by ZO-1 and ZO-2, the dual deletion of both was found to prevent the formation of TJ, ultimately leading to disorganization of the mouse embryoid epithelium and impaired barrier function [26].

TJPs modulate paracellular permeability, enabling selective molecular and ionic transport while restricting the movement of others [27,28]. In terms of intermolecular interactions, the tightness of TJs is mainly regulated by interactions between OCLN and CLDN proteins, which in turn modulate the paracellular permeability of ions and small molecules across the intestinal epithelium [29]. The ion permeability and charge selectivity in TJs vary among different types of epithelial cells. Research has demonstrated that CLDN-4 is directly implicated in modulating TJ permeability in cultured cells. Specifically, the overexpression of CLDN-4 has been shown to augment transepithelial electrical resistance (TEER) and diminish cationic selectivity in MDCK II cells, indicating its role in tightening the paracellular barrier and influencing ion selectivity [30]. CLDNs are major determinants of TJ permeability, and their expression patterns define diverse TJ permeability traits, while individual claudin permeability properties remain incompletely clarified due to technical limitations of overexpression/knockdown assays [31]. CLDN-2 is the best-characterized subtype, independently forming cation-selective TJ channels and determining the leaky permeability property of MDCK II cell TJs [32]. The carboxyl terminus of OCLN can undergo a state of phosphorylation of amino acid residues, the alteration of which can regulate the permeability of the TJ. Cells are also able to dynamically regulate the permeability of substances by altering the expression level or modification status of TJPs to influence inflammation, cellular value addition, or differentiation. Studies have shown [33] that OCLN is continuously distributed and highly expressed at intercellular contacts in brain endothelial cells, and the regulation of OCLN expression may be a key factor in the permeability properties of TJ in endothelial cells from different tissues.

TJPs are integral to the modulation of immune signaling, influencing immune responses through the regulation of immune cell activity and distribution, as well as cytokine permeability [22,28]. OCLN participates in intracellular signaling pathways, responds to external stimuli, and regulates the immune barrier function. When cells are stimulated by inflammatory factors, the expression and distribution of OCLN change, thereby regulating intercellular permeability to influence the immune response [34]. OCLN has been identified as a significant effector of interleukin-22 (IL-22) in preserving the intestinal barrier’s integrity. In the context of ulcerative colitis (UC), the expression of OCLN can be increased by treatment with IL-22, which in turn protects the intestinal mucosa from inflammation [35]. TJPs also influence the expression and release of inflammatory cytokines and intercellular adhesion molecules, which regulate immune cell migration and inflammatory responses. Increased expression of CLDN-2 when an inflammatory response occurs leads to increased intestinal barrier permeability and promotes immune cell infiltration and inflammatory responses. Further studies showed that protein levels of CLDN, OCLN, and ZO-1 were negatively correlated with mRNA levels of IL-6, interleukin-1β (IL-1β), and Tumor Necrosis Factor-α (TNF-α) in mice infected with bacteria [36].

## 3. Characterization of the Intestinal Barrier in Weaned Piglets

### 3.1. Changes in Intestinal Morphology and Structure

Intestinal morphology and structure are subject to dynamic alterations that are influenced by a multitude of factors, including physiological stress, nutritional intake, and microbial interactions [27,29]. Maintaining the integrity of the intestine is essential for optimizing the digestion and absorption of nutrients. However, weaning stress leads to atrophy of the intestinal mucosa, shortening of the villi, and deepening of the crypts, which reduces the area of intestinal absorption and affects the absorption and utilization of nutrients [37]. Existing studies have demonstrated that villus height (VH), crypt depth (CD), and the villus height-to-crypt depth ratio (VCR) are important indicators for assessing nutrient absorption capacity in animals [31]. A reduction in VH and VCR reflects impaired intestinal mucosal function, which further results in decreased intestinal digestive and absorptive capacities [38]. Notably, dynamic morphological observations of jejunal tissue in piglets at different time points post-weaning have revealed a marked decrease in jejunal villus height during the third week after weaning [39,40]. Moreover, a sustained reduction in VH is a direct manifestation of impaired intestinal mucosal function [41]. In general, villus atrophy and degeneration are closely associated with intestinal mucosal barrier damage, and TJPs represent the core components of the intestinal mucosal physical barrier. Mucosal barrier injury is often accompanied by a significant downregulation of TJP expression levels, which subsequently increases intestinal barrier permeability (Table 1). This not only impairs intestinal nutrient absorption but also elevates susceptibility to pathogenic infections. Therefore, monitoring the expression of intestinal tight junction proteins can further facilitate the maintenance of intestinal health in piglets.

### 3.2. Damage to the Intestinal Mucosal Barrier

Weaning in piglets can lead to significant stress, which in turn disrupts the homeostasis between intestinal mucosal epithelial cell proliferation and apoptosis. This disruption can increase intestinal mucosal permeability, facilitating the entry of bacteria, endotoxins, and other harmful substances into the bloodstream, thereby triggering a systemic inflammatory response and negatively affecting intestinal barrier function [22,23]. In the intestinal epithelial mucosal barrier, TJPs are the primary structural components that regulate the paracellular transport of solutes and maintain barrier integrity. These proteins, predominantly transmembrane proteins, play a critical role in sealing the spaces between adjacent cells and restricting the passage of hydrophilic molecules [42]. The expression levels of TJ proteins in the intestinal epithelium are pivotal biomarkers for assessing the enteromechanical barrier’s integrity. Weaning stress in piglets has been demonstrated to impair the body’s barrier functions, particularly by downregulating the expression of TJ proteins [43]. This reduction in TJ protein expression facilitates the translocation of bacteria and toxins across the intestinal mucosa, leading to the release of various cellular inflammatory factors and subsequent intestinal inflammation [44]. Studies have consistently shown that weaning stress in piglets causes a reduction in the expression of TJPs within the intestinal epithelium, leading to a decrease in the TEER and a significant increase in intestinal permeability [45]. As a result, weaning stress has a detrimental effect on the intestinal barrier function (Table 1). The compromised barrier function not only enhances the permeability of the intestinal mucosa but also triggers a systemic inflammatory response, highlighting the significance of maintaining TJ protein expression for gut health and immune response modulation in weaned piglets [42,46].

### 3.3. Imbalance of Intestinal Microecology

Weaning stress alters the structure of the intestinal microbial community in piglets, leading to intestinal microecological imbalance and affecting intestinal health [47,48]. When piglets are in the stage of lactation, bacteria such as Lactobacillus and Streptococcus are able to effectively utilize the nutrients in breast milk and are the dominant flora in the stomach and small intestine of piglets [49]. Weaning may induce an increase in intestinal pH and a marked reduction in bactericidal capacity in piglets, which in turn disrupts the gastrointestinal flora composition [50,51,52]. This leads to a decrease in beneficial gut flora (e.g., *Lactobacillus*) and an overgrowth of certain pathogenic microorganisms, including pathogenic *Escherichia coli* [53]. In addition, oxidative stress in the intestines of piglets can also lead to an imbalance of gut microbiota. Disruption of the intestinal microbiota structure in piglets is an important indicator of impaired intestinal barrier function, which can result in an increase in intestinal permeability and lead to the ectopic invasion of viruses and endotoxins. It is noteworthy that enriching beneficial gut bacteria and promoting short-chain fatty acid metabolism can effectively alleviate intestinal mucosal damage, such as improving the villus-to-crypt ratio in the intestines of piglets, though these findings are largely derived from analyses of intestinal morphological phenotypes [54]. Research has found that the balance of the gut microbiota is significantly positively correlated with the expression of TJPs [55]. Nevertheless, research investigating the direct regulatory effects of TJPs on gut microbiota composition remains scarce. Collectively, these studies indicate that intestinal barrier impairment is indeed a critical contributor to gut microbiota dysbiosis (Table 1).

### 3.4. Impaired Immune System

Weaning stress inhibits the function of the intestinal immune system of piglets; consequently, the intestinal resistance to the original pathogenic microorganisms is reduced, making them prone to diarrhea and other diseases [56,57]. Newborn piglets do not obtain protection from maternal antibodies through the placenta and rely mainly on immunoglobulin absorption from colostrum [58]. Research has confirmed that early weaning stress can induce a downregulation of digestive enzyme activity in piglets and lead to impaired pancreatic digestive enzyme function [59]. This disruption of digestive enzyme activity not only directly affects the digestion and absorption of nutrients but also adversely impacts the immune barrier function of the intestinal mucosa by disrupting the homeostasis of the gut microbiota and further impairs the expression and structural integrity of intestinal TJPs as a consequence. Changes in intestinal cytokine expression are one of the main features of intestinal inflammatory responses. Previous studies have shown that early weaning stress in piglets reduces circulating antibody levels and suppresses cellular immunity. Research has found that weaning stress impairs the immune system of piglets and induces intestinal inflammatory responses, significantly increasing the gene expression of IL-6, TNF-α, and interferon γ (IFN-γ) in the intestine [60,61]. In addition, weaning stress in piglets can lead to significant dysregulation of the expression levels of CLDN-1, OCLN, ZO-1, ZO-2, and ZO-3. Further analysis suggests that the underlying mechanism may involve mitogen-activated protein kinases (MAPKs) blocking the phosphorylation of extracellular regulated protein kinases-1 (ERK1)/ERK2, thereby increasing intestinal epithelial solute permeability and altering the localization and phosphorylation of tight junction proteins, ultimately compromising the integrity of the intestinal epithelial tight junction structure [62]. Studies on inflammatory models of piglet intestines have shown that supplementation with glutamine can inhibit the activation of the TLR4-p38/MAPK-nuclear factor kappa-B (NF-κB) pathway, reducing intestinal mucosal damage and inflammation [63]. During early weaning, the fragile state of enzyme activity imbalance and weakened immune function may exacerbate the dysregulation of TJPs through pathways such as MAPK. Therefore, maintaining the integrity of TJPs is crucial for mitigating the systemic impact of immune-physiological changes during weaning (Table 1).

## 4. The Role of TJPs in the Intestinal Barrier of Piglets + 6

### 4.1. Maintenance of Intestinal Cell Permeability

**Figure 2:**
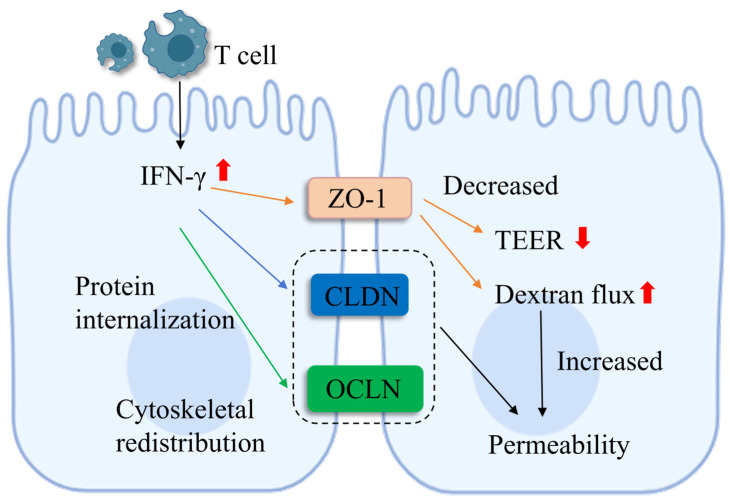
**Mechanisms of TJPs in regulating intestinal cell permeability.** Under inflammatory stress, such as IFN-γ exposure, TJPs like OCLN and CLDN undergo redistribution and internalization, leading to a decrease in transepithelial electrical resistance (TEER) and increased paracellular flux of pathogens. OCLN: occludin; CLDN: claudins; ZO-1: zona occludens-1; IFN-γ: interferon-gamma.

In the piglet intestine, TJPs maintain normal intestinal paracellular permeability and protect piglets from intestinal diseases such as diarrhea by occluding paracellular spaces between intestinal epithelial cells and preventing pathogenic microorganisms and harmful substances from translocating across the intestinal epithelium [64,65]. Piglet intestinal permeability refers to the ability of specific molecules to diffuse across the intestinal epithelial barrier in a non-carrier-dependent manner, and intestinal paracellular permeability is closely associated with intestinal mucosal barrier function [66]. Studies have indicated that various intestinal diseases lead to increased intestinal paracellular permeability; conversely, elevated intestinal paracellular permeability may compromise intestinal mucosal barrier function, thereby inducing the onset of multiple intestinal diseases [67,68]. Epithelial TJPs are the key determinants of intestinal mucosal barrier paracellular permeability. Studies have shown that the expression of CLDN-1 and OCLN was significantly reduced in piglets fed a high-fat diet, which may result in increased intestinal paracellular permeability [69]. Interferon-gamma (IFN-γ) is a pro-inflammatory cytokine predominantly secreted by T cells. IFN-γ disrupts TJPs (OCLN, CLDN-1/2/4, ZO-1) via downregulation, cleavage, and internalization, leading to increased intestinal permeability and barrier dysfunction [70]. In piglets, abnormal IFN-γ elevation induces intestinal tight junction damage and elevated permeability, increasing susceptibility to intestinal diseases (for example, PEDV infection and diarrhea) [71]. Studies have demonstrated [72] that LPS-induced intestinal injury downregulates the expression of these key TJPs in the jejunum, which results in reduced TEER and increased intestinal paracellular permeability in piglet intestinal epithelial cells. Conversely, this also precisely demonstrates that tight junction proteins are crucial for maintaining intestinal permeability in piglets. ZO proteins are integral to the regulation of intestinal TJs and the permeability of intestinal epithelial cells. Furthermore, the upregulation of TEER is associated with an increase in ZO proteins [73]. Studies have also found [74] that high doses of dietary SBAs (0.1–0.2%) reduce the expression of two tight junction proteins, occludin and ZO-1, in piglet intestinal epithelial cells, thereby increasing intestinal permeability, indicating that the expression of tight junction proteins has a significant effect on intestinal permeability in piglets. Enhancing TJP expression through nutritional regulation and maintaining normal intercellular permeability is beneficial for preserving intestinal barrier function in piglets [75,76]. Studies have shown that caffeic acid regulates the expression of colonic microbiota and tight junction proteins in weaned piglets, which alleviates intestinal inflammation, maintains normal intestinal permeability, and improves intestinal barrier function [77]. TJPs are the key regulators of intestinal permeability in pigs, and stress-induced TJP dysfunction directly elevates intestinal permeability in pigs [78,79]. Due to the immature and incomplete intestinal tract development of early-weaned piglets, external stress can induce tight junction damage and increased intestinal permeability, leading to intestinal dysbiosis and growth impairment [80,81]. Therefore, the structural integrity and expression level of TJs play a crucial role in maintaining normal intestinal permeability and exert core effects in intestinal barrier protection and permeability regulation for piglet intestinal health (Figure 2).

### 4.2. Improvement of the Intestinal Immune Barrier

**Figure 3:**
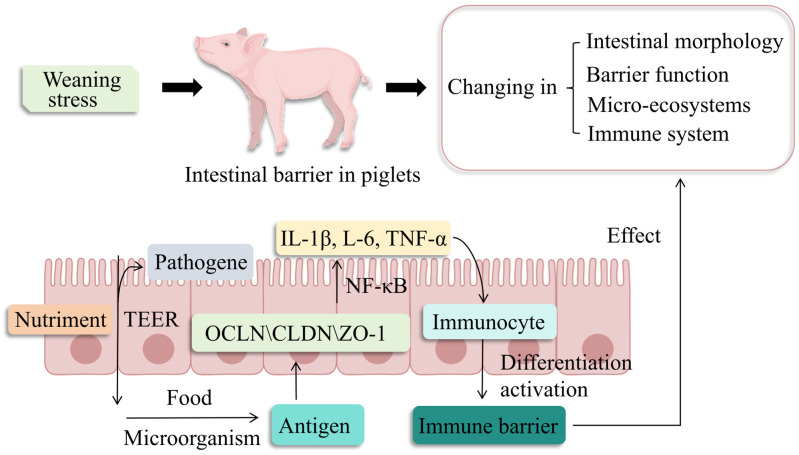
**The role of TJPs in the intestinal immune barrier.** Intact TJPs can prevent the transport of antigens such as microbes and food from the lumen. Dysregulation of OCLN and CLDN can activate immune signaling pathways such as NF-κB, triggering the release of pro-inflammatory cytokines like TNF-α and IL-6, which affect the intestinal barrier, immune capability, and microbial diversity in piglets. OCLN: occludin; CLDN: claudins; ZO-1: zona occludens-1; IL-1β: interleukin-1β; IL-6: interleukin-6; TNF-α: tumor necrosis factor α; TEER: transendothelial electrical resistance.

TJPs may improve intestinal immune barrier function by regulating intercellular signaling, which could promote intestinal immune cell differentiation and activation and enhance resistance of piglets to pathogens [82,83]. Intestinal epithelial cells sense luminal microbial and dietary antigens via TJP-mediated barriers and may release cytokines (IL-1β, IL-6, IL-8, TNF-α) in response to potential threats (Figure 3, illustrating the TJP-dependent antigen-sensing cascade triggering immune cytokine secretion). These signaling molecules may enhance the antimicrobial activity of macrophages and lymphocytes by promoting immune cell activation [84]. In piglet intestinal epithelial cells, the intact intestinal barrier formed by OCLN and CLDN between cells helps to reduce the inflammatory response and provides a stable microenvironment for immune cell differentiation and activation [85]. Different types of CLDN proteins determine the selective permeability of TJs, and the expression and distribution of CLDN in the piglet intestine can influence substance absorption and pathogen defense, thus indirectly affecting immune cell activation and signaling [86,87]. Rac1 is critical for intestinal epithelial junction assembly and TJ barrier integrity during inflammation. ETEC K88 is associated with suppressed CaSR/Rac1/PLC-γ1 signaling and reduced TJP expression, which may induce intestinal barrier injury and inflammation (elevated IL-8, TNF-α). In contrast, tryptophan supplementation may enable porcine TJPs (OCLN, ZO-1, CLDN-1) to modulate intestinal inflammation via the CaSR/Rac1/PLC-γ1 pathway [88]. It was found that upregulated ileal ZO-1 expression in piglets may correlate with increased NF-κB, IL-6, and TNF-α levels; OCLN and p-NF-κB expression also tended to increase, which may collectively reduce diarrhea and improve immune status and growth performance in weaned piglets [89]. Pro-inflammatory factors (TNF-α, IFN-γ) may alter TJP expression via immune signaling activation, potentially inducing intestinal epithelial apoptosis and inflammation. For instance, IFN-γ may induce claudin internalization from TJ regions, which is associated with decreased TEER and increased paracellular permeability [90]. Intestinal mucosal damage in piglets may activate local immune responses, leading to elevated pro-inflammatory cytokines, dysregulated TJPs (OCLN, CLDN, ZO-1) and signaling pathways, epithelial barrier disruption, and subsequent impaired intestinal immune function [91]. Interestingly, TJPs (OCLN, ZO-1) can coordinate with the NLRX1/ERK/MLC signaling pathway to suppress upstream pathogenic signal activation and block downstream barrier-disruptive pathways, thereby maintaining TJ integrity, enhancing intestinal immune barrier function, and resisting PAstV-4-induced porcine intestinal mucosal injury, which is beneficial to protecting pig intestinal health [92]. Therefore, the interactions between TJPs, immune signaling molecules, and pathways may play a crucial role in maintaining the intestinal immune barrier in piglets. Although related studies have confirmed that regulating immune signaling pathways and TJP expression can improve the intestinal barrier, the specific mechanisms by which TJPs affect the intestinal immune capacity of piglets remain insufficiently understood due to the differences in animal models and physiological backgrounds across existing studies.

### 4.3. Regulating the Balance of Intestinal Flora

**Figure 4:**
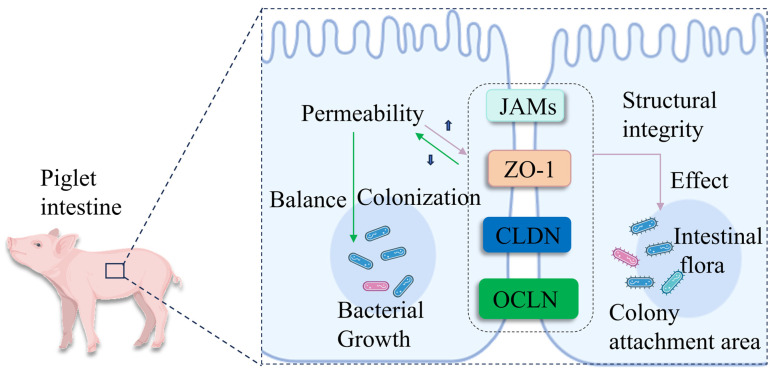
**Interactions between TJPs and the gut microbiota.** In the figure, TJPs reduce intestinal epithelial permeability and alleviate epithelial damage by maintaining ZO-1 phosphorylation levels, enhancing OCLN activity, and upregulating the expression of OCLN, ZO-1, and CLDN, thereby preserving barrier integrity. Consequently, high expression of TJPs provides a stable environment for the colonization of beneficial bacteria, ensuring gut microbiota balance. In contrast, the loss of OCLN and CLDN can promote the adhesion of pathogens such as *Escherichia coli*, leading to gut microbiota dysbiosis. OCLN: occludin; CLDN: claudins; JAMs: junctional adhesion molecules; ZO-1: zona occludens-1.

TJPs may influence intestinal microbiota colonization and proliferation and help maintain microbial homeostasis by regulating epithelial paracellular permeability; TJP integrity is associated with improved microbial adhesion in the piglet intestine [87,93]. OCLN and ZO-1 are key TJ complex components, and their reduced expression may impair intestinal barrier function, increase permeability, and alter intestinal microbial structure and function [94]. Weaning stress (environmental/dietary changes) may induce intestinal microbial shifts in piglets, while preserved ZO-1 phosphorylation and upregulated OCLN, ZO-1, and CLDN expression may reduce permeability, alleviate epithelial damage, and preserve barrier integrity. These changes may support normal microbial colonization and sustain intestinal homeostasis in piglets (Figure 4 illustrates the mechanistic link between TJP expression, intestinal barrier integrity, and microbial balance in stressed weaned piglets). Upregulated OCLN may also maintain microbial homeostasis, and probiotics may promote TJP expression to modulate intestinal immunity, improve microecology, and enhance mucosal barrier function, with potential anti-inflammatory and immunomodulatory roles in piglet intestinal inflammation [6,95]. Previous studies have suggested that Lactobacillus lactis may improve intestinal damage tolerance by inducing the expression of small proline-rich protein 2A (Sprr2A) in piglet intestinal villi and upregulating ZO-1 and OCLN to promote TJ integrity, which may reduce epithelial apoptosis and modulate piglet intestinal microbiota [96]. TJPs may also regulate bacterial adhesion and permeability by modifying epithelial intercellular gap size. It is worth noting that tight junction proteins (OCLN, CLDN, and ZO-1) are positively correlated with beneficial microbiota colonization in neonatal piglets. GMF upregulates their gene expression to facilitate the colonization of Lactobacillus and other beneficial genera, stabilize intestinal flora structure by enhancing barrier function, and further promote intestinal development [97]. ZO proteins may anchor TJ-associated proteins to the cytoskeleton to maintain TJ integrity, which is linked to successful microbial colonization in weaned piglets [98]. Altered transmembrane TJ protein expression may affect intestinal microbial composition. Studies have found that reduced OCLN/CLDN expression may increase pathogenic bacterial infestations (for example, ETEC) and alter microbial diversity [99]. Related studies have found that enhancing the functional integrity of the intestinal mucosal barrier in piglets can further improve the intestinal microecological environment of piglets [100]. Dysregulated OCLN, CLDN, and ZO-1 expression may reduce intestinal resistance to pathogens, potentially leading to microbial dysbiosis [90]. Although previous studies have already confirmed that the expression of TJPs is positively correlated with the structure and abundance of the microbiota, indicating that maintaining TJP integrity exerts beneficial effects on the gut microbiota of piglets. However, current research in this field still has certain limitations. Most evidence is based on correlation rather than clear causal mechanisms between TJPs and microbial colonization in piglets. Moreover, the specific regulatory effects of different TJP subtypes on distinct microbial taxa remain unclear, and further research is still needed in the future.

## 5. Conclusions

This article provides a review of the role of TJPs in the intestinal health of weaned piglets, focusing on the critical role of TJPs in the intestinal barrier of weaned piglets. It reveals their important properties in maintaining the intestinal mucosal barrier function and regulating immune responses. In addition, this review provides effective solutions to the challenges of animal intestinal stress, such as optimizing intestinal morphology and enhancing the immune barrier, thus contributing important insights to animal health biology. Further studies should explore the effects of nutritional regulation on the expression of TJPs and intestinal health and elucidate their roles in the mechanisms of intestinal diseases, which could promote the research and development of novel feed additives and nutritional strategies.

## Figures and Tables

**Table 1:** Effects of weaning stress on the expression and function of major intestinal TJPs in piglets.

Tight Junction Protein	Expression Pattern Under Weaning Stress	Core Biological Functions	Mechanistic Link to Intestinal Health Impairment
OCLN (Occludin)	Downregulated	Core transmembrane protein sealing intercellular gaps. Maintains intestinal epithelial barrier integrity. Regulates paracellular permeability of hydrophilic molecules.	Downregulation increases intestinal permeability → promotes translocation of bacteria/endotoxins → triggers mucosal inflammation → exacerbates microecological imbalance.
CLDN-1 (Claudin-1)	Downregulated	Key component of the tight junction strand network. Controls selective paracellular transport of ions and small molecules. Reinforces the structural stability of the epithelial barrier	Reduced expression impairs barrier selectivity → elevates transepithelial permeability (decreased TEER) → enhances pathogenic bacterial invasion → disrupts intestinal microecology.
CLDN-2 (Claudin-2) CLDN-4 (Claudin-4)	Not directly reported in text; inferred based on TJP family characteristics	Strengthens the tight junction barrier function by inhibiting paracellular diffusion. Interacts with other TJPs to maintain junctional integrity.	Downregulation weakens barrier tightness → facilitates translocation of luminal antigens → activates mucosal immune responses → promotes pro-inflammatory cytokine secretion (IL-6, TNF-α, IFN-γ).
ZO-1 (Zonula Occludens-1)	Downregulated	Scaffold protein linking transmembrane TJPs to the cytoskeleton. Stabilizes tight junction structure and regulates protein localization. Mediates signaling pathways involved in barrier maintenance.	Reduced expression disrupts TJP-cytoskeleton interactions → impairs tight junction assembly → enhances intestinal permeability → suppresses beneficial microbiota colonization and promotes pathogen overgrowth.

## Data Availability

No new data were created or analyzed in this study. Data sharing is not applicable to this article.
